# Bacterial Communities of Ballan Wrasse (*Labrus bergylta*) Eggs at a Commercial Marine Hatchery

**DOI:** 10.1007/s00284-020-02286-8

**Published:** 2020-11-23

**Authors:** Aileen Bone, Michaël Bekaert, Athina Papadopoulou, Stuart McMillan, Alexandra Adams, Andrew Davie, Andrew P. Desbois

**Affiliations:** Institute of Aquaculture, Faculty of Natural Sciences, University of Stirling, Stirling, FK9 4LA UK

## Abstract

Ballan wrasse (*Labrus bergylta*, Ascanius 1767) are cleaner fish cultured in northern Europe to remove sea lice from farmed Atlantic salmon (*Salmo salar*, Linnaeus 1758). Despite increasing appreciation for the importance of the microbiota on the phenotypes of vertebrates including teleosts, the microbiota of wrasse eggs has yet to be described. Therefore, the aim of this present study was to describe the bacterial component of the microbiota of ballan wrasse eggs shortly after spawning and at 5 days, once the eggs had undergone a routine incubation protocol that included surface disinfection steps in a common holding tank. Triplicate egg samples were collected from each of three spawning tanks and analysis of *16S rRNA* gene sequences revealed that 88.6% of reads could be identified to 186 taxonomic families. At Day 0, reads corresponding to members of the Vibrionaceae, Colwelliaceae and Rubritaleaceae families were detected at greatest relative abundances. Bacterial communities of eggs varied more greatly between tanks than between samples deriving from the same tank. At Day 5, there was a consistent reduction in *16S rRNA* gene sequence richness across the tanks. Even though the eggs from the different tanks were incubated in a common holding tank, the bacterial communities of the eggs from the different tanks had diverged to become increasingly dissimilar. This suggests that the disinfection and incubation exerted differential effects of the microbiota of the eggs from each tank and that the influence of the tank water on the composition of the egg microbiota was lower than expected. This first comprehensive description of the ballan wrasse egg bacterial community is an initial step to understand the role and function of the microbiota on the phenotype of this fish. In future, mass DNA sequencing methods may be applied in hatcheries to screen for pathogens and as a tool to assess the health status of eggs.

**Electronic supplementary material:**

The online version of this article (10.1007/s00284-020-02286-8) contains supplementary material, which is available to authorized users.

## Introduction

Ballan wrasse (*Labrus bergylta* Ascanius 1767) are cleaner fish recognised as an established and important biological component of sea lice control in Atlantic salmon (*Salmo salar* Linnaeus, 1758) farming in northern Europe [1–3] The cleaner fish remove sea lice from infested salmon and recent efforts have reduced reliance on wild capture, with more than half of demand for cleaner fish now delivered from farmed origins [4, 5].

Health management and infection control are of central importance in aquaculture. During ballan wrasse production, eggs are exposed to broad-spectrum disinfectants, such as formalin and bronopol, in an effort to inactivate potential pathogens attached to or within the egg that may hinder or prevent development and hatching and may impact the health of larvae thereafter [6, 7]. Disinfection is effective for improving egg survival but indiscriminate and it likely alters the entire microbial community (i.e. microbiota) of the egg [8]. However, it is increasingly apparent that the microbiota of vertebrates, including teleosts, can profoundly affect many aspects of an organism’s development, health status, metabolic capability, behaviour, and other phenotypes [9–11]. Greater understanding of the microbiota of fish eggs and its effects on various traits is required. Ultimately, the ability to manipulate the microbiota may deliver early-life stage improvements in survival and development, as well as benefits at later life stages deriving from phenotypes such as enhanced immune protection and better feed conversion [8, 9, 12, 13].

After hatching, bacteria are introduced into the sterile gastrointestinal tract of the developing larva from the water and through consumption of egg chorion material, and this contributes to the establishment and development of the internal microbiota [14, 15]. Some maternal-derived bacteria can even exert beneficial effects on egg development by preventing the attachment of potential pathogens [13, 16]. Despite this, little is known of the composition of the microbiota of marine fish eggs, including those of ballan wrasse; however, recent advances in DNA sequencing technology permit detection and taxonomic classification of almost all bacteria in a sample, and not just those that can be cultured, through sequencing of partial segments of the 16S ribosomal RNA (rRNA) gene. Such methodologies provide novel insights into the dynamic flux of microbiota communities that can be utilised in a range of medical and agricultural applications, including disease prevention, diagnosis and treatment [10, 17, 18]. Still, these techniques have yet to be widely applied in the context of aquaculture hatchery management where microbial community management has been a long-standing challenge [8, 12].

Therefore, the aim of this present study was to apply a mass DNA sequencing approach to characterise the bacterial component of the microbiota of ballan wrasse eggs during commercial production, in samples collected shortly after spawning and at 5 days, once these had undergone a routine incubation protocol that included surface disinfection steps.

## Materials & Methods

### Collection of Egg Samples

**Fig. 1:**
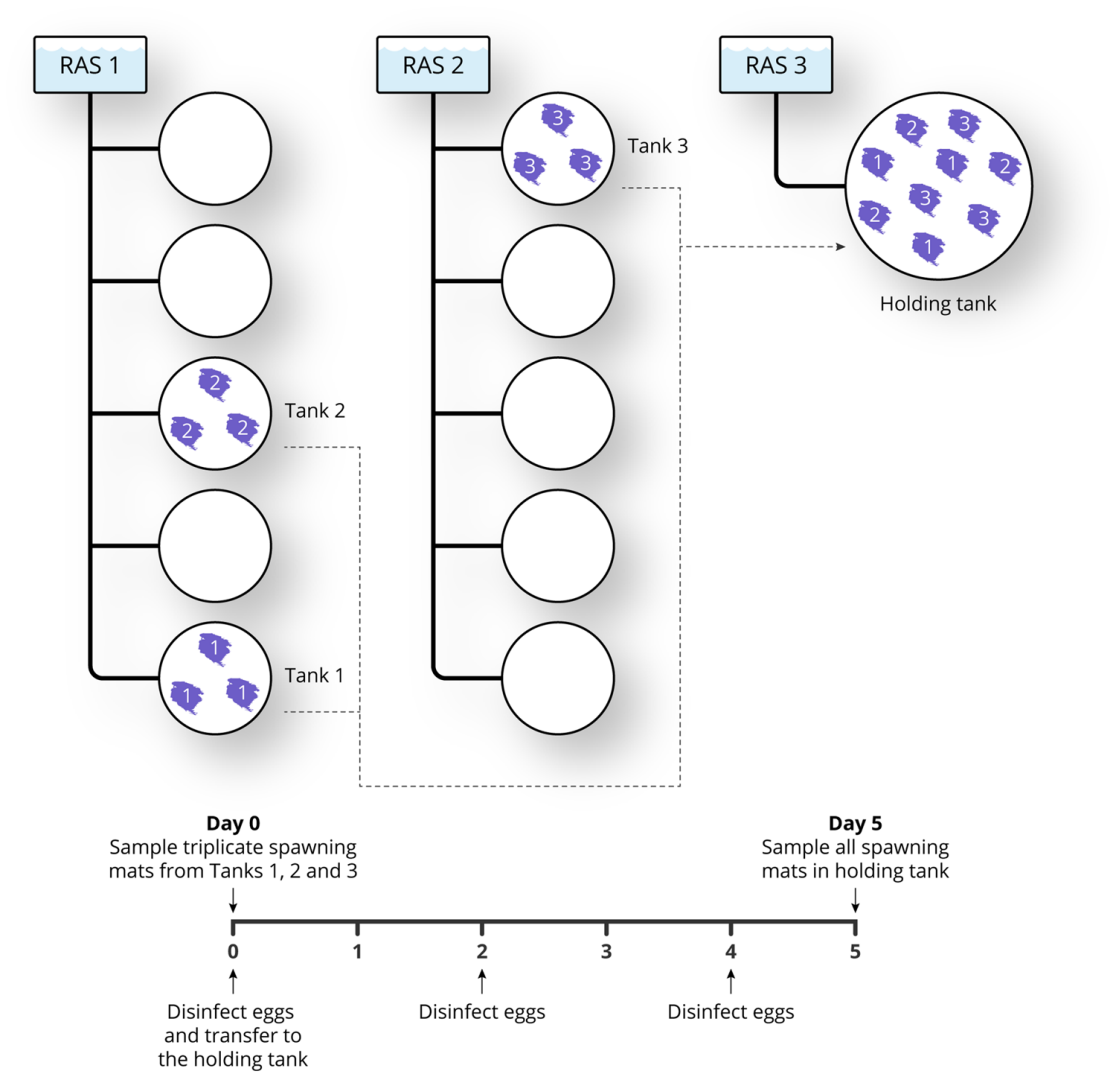
Schematic representation of the experimental design. At Day 0 (i.e. within 24 h of spawning), ballan wrasse eggs were collected from each of three spawning mats from three separate spawning tanks (1 g of eggs from each mat). The ten spawning tanks at the site are connected as two banks of five to separate recirculation systems (RAS), with Tanks 1 and 2 sharing the same RAS. The eggs remaining on each mat underwent surface disinfection by bathing in formalin (100 ppm) for 1 h and then bronopol (100 ppm) for 1 h, before the mats were transferred to a common holding tank connected to a third RAS. On Day 2 and Day 4, the surface disinfection steps were repeated, and the mats were returned to the common holding tank. At Day 5, a further 1 g of eggs were collected from each mat

Ballan wrasse eggs were collected within 24 h of spawning (‘Day 0’) from each of three spawning mats from three spawning tanks at a commercial ballan wrasse hatchery in Machrihanish, Scotland, giving nine samples in total. Each broodstock tank was 7 m^3^ and these were connected as banks of five to a common recirculation system (TMC 10000 recirculation system; Tropical Marine Centre, Chorleywood, UK), operating at a 2-h turnover rate. The recirculation system was equipped with protein skimmer, mechanical filtration (1000 µm), biofilters, ultraviolet disinfection and thermal control to maintain a constant water temperature of 12 °C. Study Tanks 1 and 2 were connected via a common recirculation unit, while study Tank 3 was part of another unit. Typically, in a given day the mats from the same tank will derive from a single pair mating [19]. Each sample consisted *ca.* 1 g of eggs that were scraped carefully into a universal bottle, frozen immediately in a dry shipper and then stored at − 70 °C. Each mat and the rest of the eggs thereon underwent surface disinfection by bathing in formalin (100 ppm; Sigma Aldrich, Gillingham, UK) for 1 h followed immediately by bathing in bronopol (100 ppm; Pyceze, Novartis Animal Vaccines Ltd, Litlington, UK) for 1 h, and then the mats and remaining eggs were incubated at 12–13 °C in a common holding tank connected to a third independent recirculation system (TMC 5000 recirculation system; Tropical Marine Centre). On Day 2 and Day 4, the mats were removed from the common holding tank and the surface disinfection steps were repeated, before returning the mats to the same holding tank. At Day 5, eggs were collected from each mat as described above (a further nine samples, giving 18 in total). The egg volumes did not change noticeably (by visual inspection under a light microscope) during the 5-day incubation. A schematic diagram of the experimental design is shown in Fig. 1.

### DNA Extraction

DNA was extracted from *ca.* 200 mg wet weight of each frozen egg sample (which included the gum layer) using the QIAamp Fast DNA Stool Mini Kit according to the manufacturer’s instructions (QIAamp Fast DNA Stool Mini Handbook, 2014; available at: www.qiagen.com). Each sample was defrosted and then 1 mL of InhibitEX buffer and at least five sterile glass beads was added before placing in a bead beater for 2 min. At the end of the extraction procedure, the DNA in each sample was eluted in 200 µL ATE buffer. A buffers-only control was performed and included in all following procedures.

### Quantification of dsDNA

Total dsDNA in each sample was quantified by fluorimetry (Qubit 2.0; Life Technologies, Paisley, UK) using broad range (BR) reagents. To each of 19 0.5-mL microcentrifuge tubes (Axygen; Tewksbury, MA, USA) was added 95 µL of 1:200 BR dye:buffer mastermix and 4 µL tris–EDTA buffer (pH 8) with 1 µL of test sample (for the 18 experimental samples) or 1 µL control solution (for the buffers-only control); two standards were prepared (95 µL of 1:200 BR dye:buffer mastermix and 5 µL of either BR standard) for calibration. The samples were incubated at room temperature for 5 min before being read. Hereafter, high sensitivity reagents only were used to quantify total dsDNA in samples.

### Preparation of 16S rRNA Libraries with Adaptors

The *16S rRNA* libraries were prepared for sequencing on a MiSeq platform according to manufacturer’s instructions [20] using primers (Eurofins, Brussels, Belgium) designed to amplify the V3-V4 hypervariable region of the *16S rRNA* gene, with some minor modifications to the protocol. Notably, Q5 Hot Start High-Fidelity DNA Polymerase (New England Biolabs, Ipswich, UK) was used, and when amplifying *16S rRNA* sequences from each sample the reaction was divided equally between three tubes to reduce PCR bias during the 25-cycle run and then re-combined for clean up using AxyPrep magnetic beads (Axygen). A no template control (NTC) was included.

### Gel Electrophoresis

Following clean up, the concentration of dsDNA was determined as above. Approximately 10 ng of total PCR product was run through a 1% agarose gel (0.5 × TAE, 0.08 µg/mL ethidium bromide) to confirm expected amplicon size (530 bp) and the presence of a single product band. A 100-bp DNA ladder was used as a molecular mass marker. The NTC and a non-purified but amplified sample were used as controls. The gel was run at 10 V/cm until the ladder had migrated and separated sufficiently.

### Addition of Indices and Sequencing Primer

The Nextera XT Index kit (Illumina) was used to uniquely index each of the PCR amplified samples, following the Nextera Low Plex Pooling Guidelines [21]. The resultant indexed amplicons were cleaned up as above and the final sample was resuspended in 27.5 µL of 10 mM Tris (pH 8.0). Purified PCR products were quantified and run through a 1% agarose gel as above to confirm expected amplicon size (601 bp) and the presence of a single product band.

### Sequencing on MiSeq Platform

Each sample was adjusted to 20 nM in 10 mM Tris (pH 8.0) and then pooled in equal volumes to give a final library sample. The dsDNA concentration in this library was determined as above, and prepared for sequencing according to the standard protocol [20]. The library (final concentration of 4 pM and including an 8% phiX spike-in) was run on a MiSeq sequencer (250 base paired-end reads; 500-cycle v2 reagent kit).

### Analysis and Processing of Sequencing Data

The mothur v1.42.0 [22] was used to analyse the sequencing data following the MiSeq standard operation procedure of Schloss et al. [23]. Briefly, all paired-end sequences were combined, sequence reads were aligned and taxonomically classified with the SILVA database release 132 [24], and chimeric sequences removed by applying VSEARCH v2.9.1 [25]. Unclassified sequences were clustered to operational taxonomic units (OTUs) at a distance cut-off level of 3%. OTUs with no match in the SILVA database were grouped together as “unclassified”. Taxonomic assignments determined at the family level were used in subsequent analyses (Supplementary Table S1), although some OTUs that did not match at family level were reported at a higher taxonomic level (i.e. order). The raw sequences are available at EBI European Nucleotide Archive database under Project PRJEB30278.

### Statistics

OTU abundances were normalised using variance-stabilising transformations and Binomial-Beta models [26]. Diversity indices, α (richness), γ (total diversity) and β (overlap; [γ/α] − 1), were calculated with the R/vegan package v2.5-3 [27] in R [28] and R/vegan v3.5.1 was used for the permutational multivariate analysis of variance (*adonis* function), and the ordination of the redundancy analysis (*rda* function after log-relative transformation). Correlation assessments were calculated with Kendall’s *tau* model [29] and *P*-values when comparing for significant differences between the Day 0 and Day 5 samples after log-relative transformation were adjusted for multiple comparisons according to Benjamini and Hochberg [30]. Evaluation of richness variation was performed using a unilateral Welch’s two sample t-test [31].

## Results

The *16S rRNA* libraries prepared from the ballan wrasse egg samples generated 14,571,037 paired-end reads (809,502 ± 94,024 per egg sample; mean ± standard deviation), excluding the 17,326 reads from the buffers-only control. A summary of the sequencing statistics is presented in Supplementary Table S2. After filtering for quality, correct amplification fragment length and chimeric sequences, 9,768,510 paired-end reads remained. Of these, 88.6% of reads were identified to 186 taxonomic families (Table 1), while the remaining 11.4% of sequences corresponded to 55 OTUs classified at the taxonomic family level (γ-index = 241) but that lack formal family names. To minimise information loss while permitting meaningful interpretation of results, we focused our analyses on family level OTUs.

**Table 1:** Alignment of all filtered *16S rRNA* reads to different taxonomic levels in libraries prepared from 18 ballan wrasse egg samples collected before disinfection (Day 0) and after disinfection and incubation (Day 5)

Taxon	OTUs	Number (classified)	Aligned reads (%)
Kingdom	1	1	100
Phylum	28	27	98.90
Class	54	45	98.36
Order	143	120	91.44
Family	241	186	88.64
Genus	494	319	59.89

In total, 88.6% of reads corresponded to 241 operational taxonomic units (OTUs) which included 186 taxonomic families with formal names

**Fig. 2:**
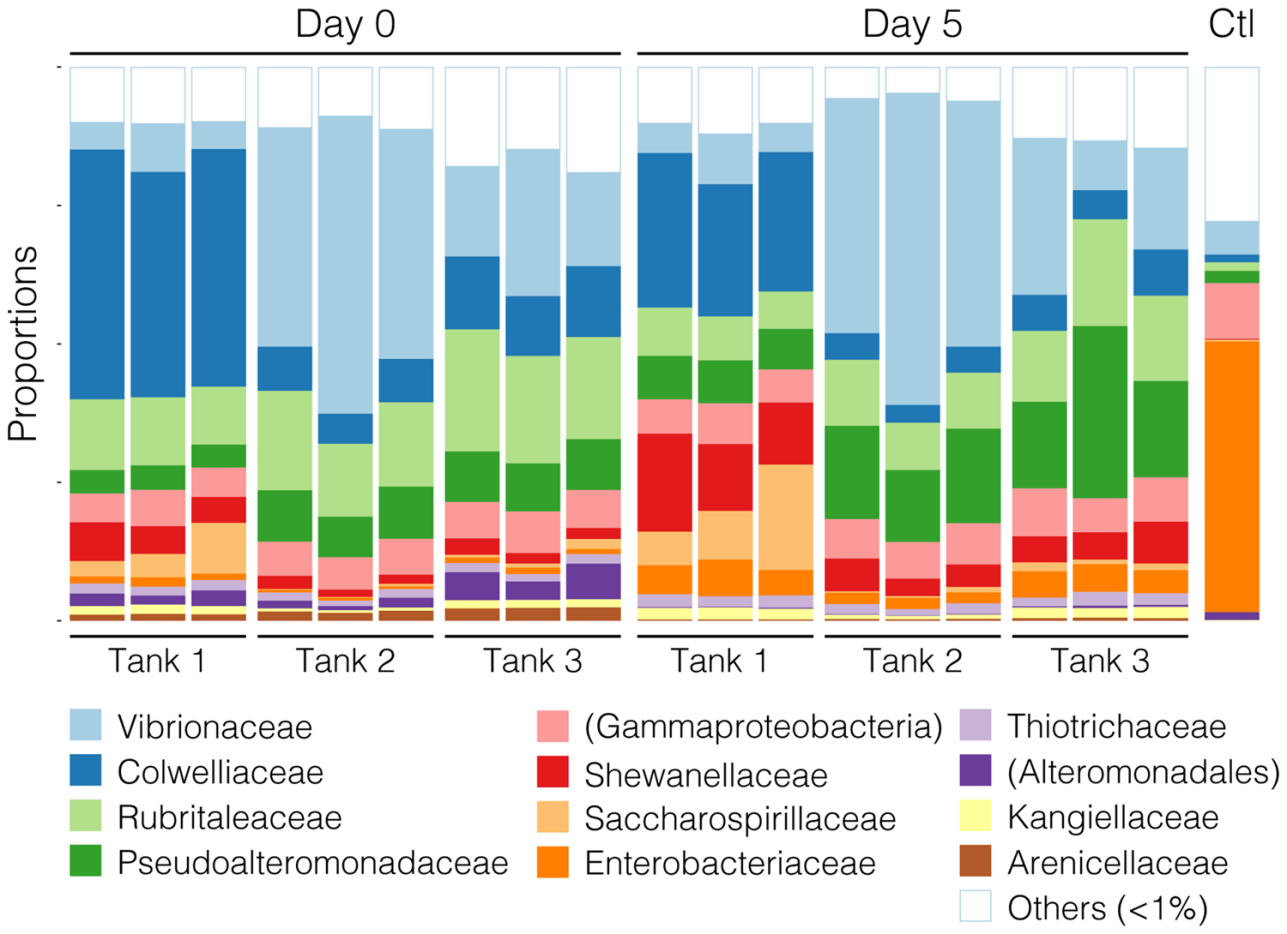
Stacked bar chart of relative composition of bacterial taxonomic families in the triplicate egg samples collected from each of three spawning tanks as determined by relative abundance of *16S rRNA* reads. After spawning (Day 0), the variation in relative composition of the bacterial communities was smaller between the samples collected from the same tank than between samples collected from different tanks. At Day 5, again, the variation in relative composition of the bacterial communities was smaller between the samples collected from the same tank than between samples collected from different tanks; however, the bacterial communities from the different tanks had diverged to be even more distinct from each other. A buffers-only control (Ctl) sample was included. Names in brackets indicate unassigned family members belonging to the named class or order

At Day 0, 223 families (and OTUs at family level) were detected with members of the Colwelliaceae (gamma-proteobacteria), Vibrionaceae (gamma-proteobacteria) and Rubritaleaceae (BV4 phylum) present at greatest relative abundance, corresponding to 23.6 ± 5.9%, 20.8 ± 5.6% and 15.8 ± 1.3% of total filtered reads, respectively (Fig. 2). Bacterial communities of the egg samples varied more between the tanks (β-index = 0.71; Adonis test: R^2^ = 0.86, *P* < 0.001) than between samples deriving from within the same tank (β-indices: Tank 1 = 0.30; Tank 2 = 0.31; Tank 3 = 0.30; Adonis test, R^2^ = 0.03, *P* = 0.311).

**Fig. 3:**
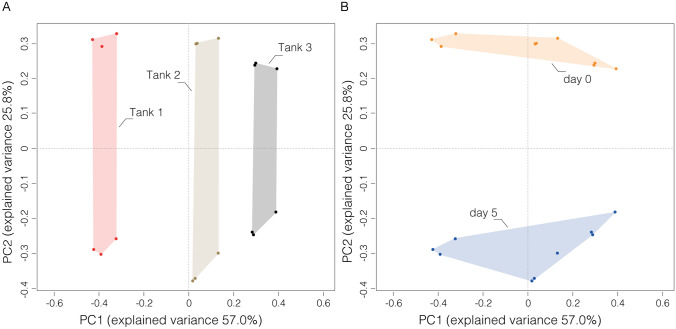
Ordination result of redundancy analysis of relative compositions of bacterial communities in triplicate egg samples collected from each of three tanks as determined by relative abundance of *16S rRNA* reads with data grouped by tank (**a**) and sample time (**b**), showing that sampling time (Day 0 vs. Day 5) had a greater effect on bacterial community composition than tank from which the samples were derived initially

At Day 5 (after routine incubation with surface disinfection steps in the common holding tanks), the bacterial communities varied more between the tanks from where they originally derived (β-index = 0.71; Adonis test: R^2^ = 0.77, *P* < 0.001) than between samples deriving from the same tank (β-indices: Tank 1 = 0.32; Tank 2 = 0.27; Tank 3 = 0.30; Adonis test: R^2^ = 0.08, *P* = 0.045). Interestingly, the bacterial communities deriving from the different tanks changed significantly between Day 0 and Day 5 (Adonis test: R^2^ = 0.22, *P* < 0.001) and, unexpectedly, these bacterial communities had diverged from each other to become more dissimilar (Welch’s two sample t-test: Tank 1, *P* < 0.001; Tank 2, *P* = 0.012; tank 3, *P* = 0.017). At Day 5, the bacterial communities of eggs collected from Tank 1 were dominated by Colwelliaceae, Shewanellaceae (gamma-proteobacteria) and Saccharospirillaceae (gamma-proteobacteria), while Tank 2 and Tank 3 communities were dominated by Vibrionaceae, Rubritaleaceae and Pseudoalteramonadaceae (gamma-proteobacteria) (Fig. 2). Sampling time (Day 0 vs. Day 5) had a smaller influence on bacterial community composition (Adonis test: R^2^ = 0.22, *P* < 0.001) than the tank from which the samples were derived initially (Fig. 3; Adonis test: R^2^ = 0.64, *P* < 0.001), and these changes were relatively consistent across the tanks (β-indices: Tank 1 = 0.58; Tank 2 = 0.70; Tank 3 = 0.64). Moreover, there was an overall reduction in bacterial community richness between Day 0 (γ-index = 223) and Day 5 (γ-index = 166), and this observation was consistent between the tanks (mean γ-indices: Tank 1 = 165 → 140; Tank 2 = 163 → 103; Tank 3 = 184 → 136). Only 18 families (of 166 in total) were present at Day 5 that were not detected at Day 0, but all of these were at < 1% relative abundance and these may have been introduced from the bacterial community present in the common holding tank water. Notably, 75 families present at Day 0 were not detected at Day 5.

**Fig. 4:**
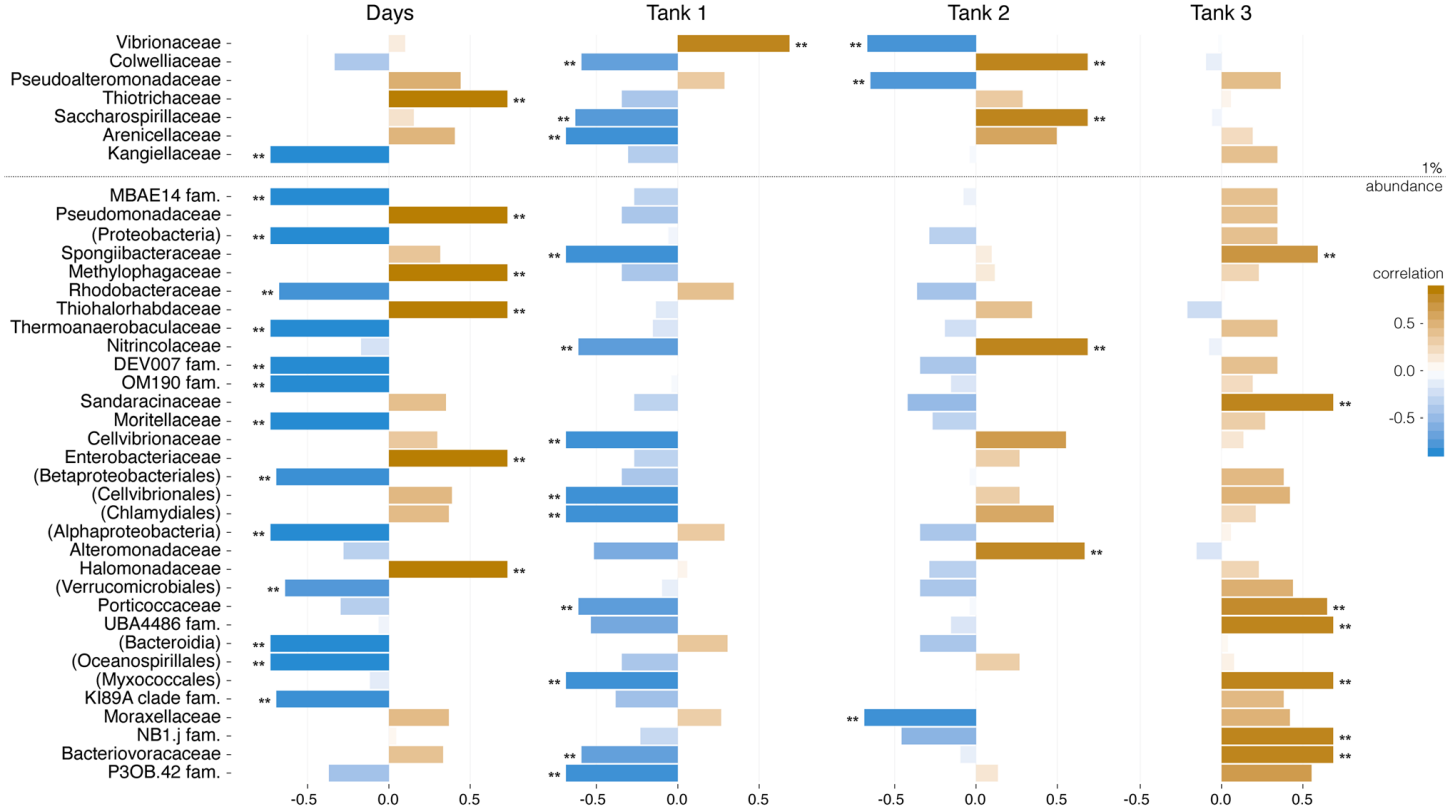
Bar chart to show correlations between changes in the relative abundance of *16S rRNA* reads corresponding to bacterial taxonomic families shortly after spawning (Day 0) and after incubation (Day 5) across all egg samples (‘Days’) and for samples derived from each of the three tanks (‘Tank 1’, ‘Tank 2’ and ‘Tank 3’). In the summative data, there was significant positive correlation in the abundance of members of the Thiotrichaceae between Day 0 and Day 5, while there was a significant negative correlation in the abundance of members of the Kangiellaceae. Nevertheless, differing and sometimes contrasting trends in relative abundances of families in the bacterial communities with time were detected between tanks, for example, the relative abundances of Vibrionaceae in samples from Tank 1 showed a significant positive correlation with time, while members of this family showed a significant negative correlation with time in Tank 2. Note that families are listed from most to least abundant and the dotted line indicates a 1% relative abundance threshold. Names in brackets indicate unassigned family members belonging to the named class or order

With respect to the taxonomic families underlying the observed changes in bacterial community compositions, overall, between Day 0 and Day 5 there was a significant positive correlation in the relative abundance of members of the Thiotrichaceae (gamma-proteobacteria), while there was a significant negative correlation in the relative abundance of members of the Kangiellaceae (gamma-proteobacteria) (Fig. 4). Various other taxonomic families at lower relative abundance (< 1%) also showed significant positive or negative correlations with time across the tanks (Fig. 4). A closer examination of the bacterial communities derived from each tank revealed differing and even contrasting trends in changes of relative abundances of the families. For example, the relative abundance of Vibrionaceae in bacterial communities deriving from Tank 1 showed a significant positive correlation between Day 0 and Day 5; however, members of this family showed a significant negative correlation in samples deriving from Tank 2, while they showed no significant change in relative abundance in Tank 3 (Fig. 4). In bacterial communities deriving from Tank 1, the relative abundance of Arenicellaceae and Saccharospirillaceae showed a significant negative correlation between Day 0 and Day 5, while in bacterial communities deriving from Tank 2 the Colwelliaceae and Saccharospirillaceae showed a significant positive correlation during this time (Fig. 4). Within the tanks, there were significant positive or negative correlations with time for various taxonomic families present at lower relative abundance (< 1%).

Finally, the raw read data were searched to identify sequences corresponding to the presence of three pathogens known to cause disease problems in the ballan wrasse hatchery and that could be present in or on the eggs, specifically *Aeromonas salmonicida* (typical and atypical subspecies), *Aliivibrio salmonicida* and *Vibrio splendidus*. The raw reads were searched because the database used did not allow for species level resolution. Across the 18 egg samples, no reads were detected that corresponded to *Aliivibrio salmonicida*, only 22 (of 9,768,510) reads corresponded to *Aeromonas salmonicida*, and just 201 reads corresponded to *V. splendidus*. Sequences corresponding to *Aeromonas salmonicida* and *V. splendidus* were detected in egg samples from each of the three tanks at Day 0, but at Day 5 *Aeromonas salmonicida* was detected only in Tank 1, while *V. splendidus* was only detected in Tank 2 and Tank 3 (Table 2). In general, there was relatively little variation in read abundances between the replicates deriving from each of the separate tanks (Table 2).

**Table 2:** Raw *16S rRNA* reads detected for three known pathogens in the triplicate egg samples collected from mats in each of three tanks in the ballan wrasse hatchery before disinfection (Day 0) and after disinfection and incubation (Day 5)

Pathogen	Day 0										Day 5										
	Tank 1			Tank 2			Tank 3			Total	Tank 1				Tank 2			Tank 3			Total
	1	2	3	1	2	3	1	2	3		1	2	3		1	2	3	1	2	3	
*Aeromonas salmonicida*	1	1	0	4	2	4	0	1	2	15	1	1	5		0	0	0	0	0	0	7
*Aliivibrio salmonicida*	0	0	0	0	0	0	0	0	0	0	0	0	0		0	0	0	0	0	0	0
*Vibrio splendidus*	1	10	1	8	24	9	7	5	2	67	0	0	0		33	56	28	4	2	11	134

No reads for these pathogens were detected in the buffers-only control

## Discussion

The increasing appreciation for the beneficial properties conferred on vertebrates by the microbiota, including roles in development, health status, immunity, metabolic capability and nutrition [9, 10, 32], has led to studies that describe the microbiota of teleost fish species at different body sites and life stages [9, 32–34]. However, far less attention has been paid to the composition of the bacterial communities of marine fish eggs [9, 12, 35], including ballan wrasse, which is cultured to remove sea lice parasites from Atlantic salmon [4]. Given that bacteria from the water and the egg itself are incorporated during the establishment of the microbiota of the developing fish larva [35, 36], certainly the composition of the early egg microbiota warrants closer scrutiny. Various environmental parameters (e.g*.* diet, water temperature, salinity and presence of antimicrobial agents) and host factors (e.g*.* species, sex, genetics, developmental stage, age) are known to influence the composition and structure of the fish microbiota [11, 13, 32, 33, 37–39], but few studies have examined changes that occur following incubation protocols in hatcheries, which include surface disinfection steps. Therefore, the aim of this present study was to describe the bacterial component of the microbiota of ballan wrasse eggs shortly after spawning and once these had undergone a 5-day incubation in a common holding tank according to the protocols at a commercial hatchery. To this end, triplicate egg samples were collected from three spawning tanks and the *16S rRNA* sequences in the DNA extracted from the eggs were analysed on an Illumina MiSeq platform.

Incubation at the hatchery for 5 days, which included surface disinfection with formalin and bronopol, led to a reduction in overall richness of the bacterial community associated with the eggs. Still, in general, the bacterial communities remained relatively unchanged between Day 0 and Day 5 in that most of the taxonomic families present shortly after spawning were also detected at Day 5 (148 of 223 families [i.e. 66.4%], with only 18 families [i.e. 8.1%] present at Day 5 that were not detected at Day 0). Indeed, families of gamma-proteobacteria, such as Vibrionaceae and Collwelliaceae, dominated the bacterial communities at Day 0 and Day 5 in this first description of the bacterial communities associated with ballan wrasse eggs. A predominance of members of the proteobacteria in the microbiota of the ballan wrasse eggs is consistent with other studies of marine hatchery fish eggs, skin and water bacterial communities [9, 14, 35, 36].

The microbiota of eggs from different mats within the same tank were relatively similar at Day 0, which was expected because the egg samples are replicates that had been exposed to the same bacterial community in the water and were derived from the same single pair mating [19]. The differences in egg microbiota composition between tanks were more noticeable, but this is not surprising given that the eggs had derived from distinct parental matings and the water microbiota likely differed between tanks. Still, given that Tanks 1 and 2 effectively shared water by being in the same recirculation system, and therefore the constituent microbiota, it is surprising that the bacterial communities of the eggs at Day 0 from these tanks were not more similar to each other than compared to the egg microbiota from Tank 3. Furthermore, the bacterial communities in the samples from the different tanks diverged further to become even more dissimilar from each other during incubation in the common holding tank between Day 0 and Day 5. This was unexpected given that the eggs for 5 days had shared the same water, and thus been exposed to the same bacterial community in the water, meaning that the egg communities might have been expected to become more similar, particularly if the water community exerted an important influence over the egg community, but this was not the case. Taken together, these observations suggest that the rearing environment (i.e. the water) has a lesser role on the egg microbiota than parental or host effects (e.g*.* direct inheritance of a microbiota, or the presence/absence of factors on or in the egg influencing the microbiota that is able to establish) [40]. This hypothesis requires further investigation but, if confirmed, it indicates that significant gains could be achieved through a deeper grasp of the microbial status of broodstock and mechanisms that may allow beneficial microbes to be passed to the offspring. The variability at Day 5 between egg batches is interesting because other studies reported the microbiota to be diverse at early-life stages before stabilising ultimately during development [32, 35, 36, 41]. Nevertheless, it remains to be determined how the composition of the ballan wrasse egg microbiota affects the microbiota at later-life stages, or how the early microbiota influences the phenotype of the developing individual. The observed composition changes could play a role in inter-individual variations in marine fish microbiota reported elsewhere (e.g*.* Uren Webster et al. [33]) and it is tempting to speculate that such divergence, possibly exacerbated by disinfection, could underlie egg batch variability in survival and development of phenotypic traits and plasticity already linked to the microbiota in other species [9, 32].

The 5-day incubation led to differential effects between tanks on relative abundances of Vibrionaceae, which contain several important fish pathogenic species. Ultimately, the purpose of egg disinfection at commercial hatcheries is to reduce microbial abundance, particularly of opportunistic pathogens, in an effort to improve embryo survival [7]. Fujimoto et al. [42] reported that lake sturgeon (*Acipenser fulvescens*, Rafinesque, 1817) eggs incubated in stream water treated with ultraviolet light and filtration showed greater survival and developed distinct bacterial communities from those incubated in untreated water. While impacts of disinfection on egg survival and the abundances of culturable bacteria were not assessed in this present study, it did show that the response of the bacterial community to disinfection was not uniform (i.e. the effects on the eggs derived from different tanks were distinct but consistent across replicates), even when the eggs were incubated in a common environment. As such, definitions of egg disinfection based on culturable microbial abundance do not fully describe the true dynamics of the microbial community changes that the present approach managed to capture. Further, the data generated in this present study were sufficiently powerful to allow the detection of selected fish pathogenic bacterial species; however, the low abundances of reads corresponding to these pathogens prohibit a firm conclusion as to whether the disinfection protocol eliminated these bacteria from the eggs, though the pathogens were detected in fewer egg samples at Day 5 than at Day 0 (Table 2). Such experiments relying on *16S rRNA* gene detection and culture could determine the effect of different disinfection protocols on particular pathogens of interest. Given that mass DNA sequencing methodologies are becoming increasingly affordable, their application within hatcheries is now feasible as a screening and/or diagnostic method to monitor microbiota stability and identify when community shifts occur that might be detrimental for production.

This present study suffers the same limitations as other studies relying on non-culture methods in that it is not possible to determine whether the *16S rRNA* gene sequences were derived from viable or metabolically active bacteria, while the PCR steps can also introduce some bias prior to sequencing [43]. Moreover, the primers used mean that mainly bacterial members of the egg microbiota were sampled herein and other microorganisms, such as eukaryotes and archaea, likely play important roles in the egg microbiota and also warrant attention [16]. The ability to describe the microbiota is an important initial step towards understanding the role of this community in the developing organism and to uncovering the functions that particular microbial species serve. As such, this present study is constrained by knowledge on these fundamental aspects of microbiota role and function, particularly in fish, though the data generated within are available publicly for re-analysis as new knowledge accumulates. Furthermore, the analyses herein were on relative abundance data and no attempt was made to quantify the bacteria in each sample, though a previous study found that the abundance of culturable bacteria changed little during 6 days of incubation under similar conditions at the same hatchery at *ca.* 10^4^ CFU per egg [44]. Follow-up studies will examine the effect on the egg microbiota of different hatchery sites and fish species, while variations observed in the bacterial communities between tanks in the present study deserve closer attention to determine the relative contribution of the tank environment (i.e. water) compared to parental transfer, which was not possible to establish fully herein. Moreover, ballan wrasse are benthic substrate spawners and the eggs are coated in a protective gum layer to ensure these adhere to the substrate upon which they are spawned [45]. In hatcheries, this gum layer containing microorganisms can be removed to enable bio-secure transfer of eggs between farming sites. Removal of the gum layer liberates the eggs from the spawning mats that have a high-organic loading and, once free, it is believed removal of the gum will increase efficacy of disinfection by separating the eggs and increasing contact with the disinfectant. However, the gum layer may play a significant role in determining microbiota composition and stability. In support of this, the DNA yield extracted from a subset of eggs exposed to enzymatic degumming was insufficient to allow the *16S rRNA* gene analyses described above (data not shown), indicating it to be the gum layer that contains the majority of the bacteria associated with the egg. Follow up experiments with eggs of other commercially important finfish species are needed to understand all possible drivers of microbiota community change in response to disinfection.

In conclusion, this is the first study to describe comprehensively the bacterial community associated with ballan wrasse eggs and once the eggs had undergone a 5-day incubation in a commercial hatchery. Few studies have investigated the microbiota of marine fish eggs, despite the potential importance of this community on the phenotype of the fish [8, 9, 35]. Further knowledge in this field may advance the efficiency of ballan wrasse culture and prove useful as diagnostic markers of egg health and, in the fullness of time, it may be possible to manipulate the egg microbiota beneficially to influence later-life traits, including improved health and welfare, disease resistance, growth rate, feed conversion and nutritional properties.

## Electronic supplementary material

Below is the link to the electronic supplementary material.

Supplementary file1 (CSV 15 KB)

Supplementary file2 (CSV 1 KB)
